# “Under the Bridge”: Looking for Ischemia in a Patient with Intramyocardial Coronary Artery Course—The Role of the Cardiopulmonary Exercise Test

**DOI:** 10.3390/jcm12175764

**Published:** 2023-09-04

**Authors:** Massimo Mapelli, Gaia Cattadori, Elisabetta Salvioni, Irene Mattavelli, Emanuele Pestrin, Umberto Attanasio, Damiano Magrì, Pietro Palermo, Piergiuseppe Agostoni

**Affiliations:** 1 Centro Cardiologico Monzino IRCCS, Via Carlo Parea 4, 20138 Milan, Italy; massimo.mapelli@cardiologicomonzino.it (M.M.); elisabetta.salvioni@cardiologicomonzino.it (E.S.); irene.mattavelli@cardiologicomonzino.it (I.M.); pietro.palermo@cardiologicomonzino.it (P.P.); piergiuseppe.agostoni@cardiologicomonzino.it (P.A.); 2 Department of Clinical Sciences and Community Health, Cardiovascular Section, University of Milan, 20122 Milan, Italy; 3 IRCCS Multimedica, 20138 Milan, Italy; 4 Unità Clinico Operativa di Clinica Medica, Università degli Studi di Trieste, Piazzale Europa, 1, 34127 Trieste, Italy; pestrin.emanuele@gmail.com; 5 Department of Translational Medical Sciences, Federico II University, Corso Umberto I 40, 80138 Naples, Italy; umberto.attanasio@yahoo.it; 6 Department of Clinical and Molecular Medicine, University “La Sapienza”, Piazzale Aldo Moro 5, 00185 Rome, Italy; damiano.magri@uniroma1.it

**Keywords:** cardiopulmonary exercise testing (CPET), myocardial bridging, stress cardiac magnetic resonance, exercise

## Abstract

Many variables obtained during cardiopulmonary exercise test (CPET), including O_2_ uptake (VO_2_) versus heart rate (HR, O_2_-pulse) and work rate (VO_2_/Watt), provide quantitative patterns of responses to exercise when left ventricular dysfunction is an effect of myocardial ischemia (MI). Therefore, CPET offers a unique approach to evaluate exercise-induced MI in the presence of fixed or dynamic coronary arteries stenosis. In this paper, we examined the case of a 74-year-old patient presenting with an ischemic CPET and a normal stress cardiac magnetic resonance (CMR) with dipyridamole. A coronary angiography demonstrated the presence of myocardial bridging (MB), a well-known congenital coronary anomaly that is able to generate MI during exercise (but not in provocative testing using coronary artery vasodilators, such as dipyridamole). Despite the good diagnostic accuracy of the imaging methods (i.e., stress CMR) in MI detection, this case shows that exercise should be the method of choice in elicit ischemia in specific cases, like MB.

## 1. Introduction

Several variables attained during the Cardiopulmonary Exercise Test (CPET), including O_2_ uptake relative to heart rate (HR, O_2_-pulse) and work rate (VO_2_/Watt), supply quantitative patterns of responses to exercise when left ventricular dysfunction is an effect of myocardial ischemia (MI) [1,2]. Specifically, O_2_-pulse is a CPET parameter that is calculated from the ratio of oxygen consumption (VO_2_) to HR. According to Fick’s law, it represents the product of stroke volume (SV) and O_2_ extraction. Given that this latter parameter (O_2_ extraction) is generally considered to be constant and predictable [3,4], the O_2_-pulse changes during a maximal ramp protocol CPET, which may be considered a metabolic surrogate of the SV variations [5,6,7]. Therefore, the development of exercise-induced MI may lead to loss of SV and a plateau (or fall) in O_2_-pulse during exercise [1]. Even if not highly specific for ischemic coronary arteries stenosis, given that these parameters (i.e., VO_2_/Watt and O_2_-pulse) also altered in other settings, such as hypertrophic cardiomyopathy [8] or dynamic mitral regurgitation, they suggest the importance of CPET in evaluating exercise-induced MI [9]. Patients with or without chest pain or dyspnea can show rapid reduction in SV as a pathological response to exercise. Myocardial bridging (MB), a congenital coronary anomaly, is a cluster of myocardial fibers crossing over the epicardial coronary arteries [10]. Although MB may be a casual finding (angiography or autopsy) or a diagnosis made in case of typical angina, patients with no symptoms may also develop MI due to dynamic systolic compression.

We present the case of a 74-year-old patient presenting with a pathological drop in O_2_-pulse and VO_2_/Watt slopes during maximal exercise and a normal stress cardiac magnetic resonance (CMR) in order to underline the importance of exercise in eliciting MI in specific cases as well as the central role of CPET in this context.

## 2. Case Description

**Figure 1:**
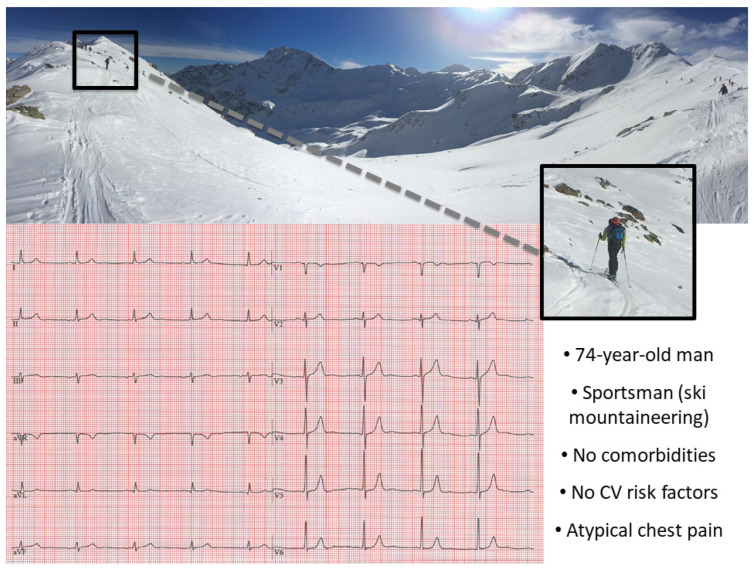
**Case presentation.** A 74-year-old man with no cardiovascular history who performs high-load physical activity (multiple ski mountaineering trips) was referred for CPET following episodes of atypical chest pain. Abbreviations: CPET = Cardiopulmonary Exercise Testing.

**Figure 2:**
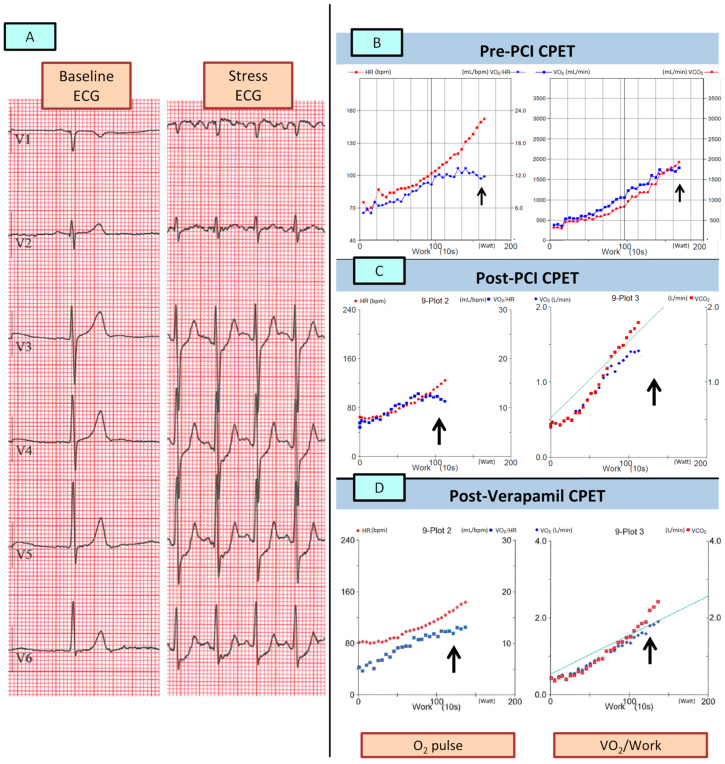
**Serial CPETs.** The figure shows the exercise-induced ECG changes (**A**) and the metabolic changes in both the baseline CPET (**B**) and in the post-PCI CPET (**C**) with a clear flattening/downsloping of the O_2_-pulse and VO_2_/Work (arrows). Panel (**D**) shows the normalization of the metabolic behavior after the introduction of Verapamil in the medical therapyAbbreviations: CPET = Cardiopulmonary Exercise Testing; PCI = Percutaneous Coronary Intervention; VO_2_ = Oxygen Intake.

A 74-year-old man, who is a sportsman (ski mountaineering) (Figure 1), was symptomatic for atypical chest pain, and underwent a maximal (respiratory exchange ratio at peak (RER) 1.1) ramp protocol CPET, showing metabolic and ECG signs of MI (Figure 2A,B). Baseline CPET data are presented in Table 1. In brief, peak VO_2_ was 27.7 mL/min/Kg (corresponding to 112% of the predicted VO_2_), peak O_2_-pulse was 11.4 mL/bpm (corresponding to 105% of the predicted peak O_2_-pulse), and maximal HR was 154 bpm (105% of the predicted maximal HR).

**Figure 3:**
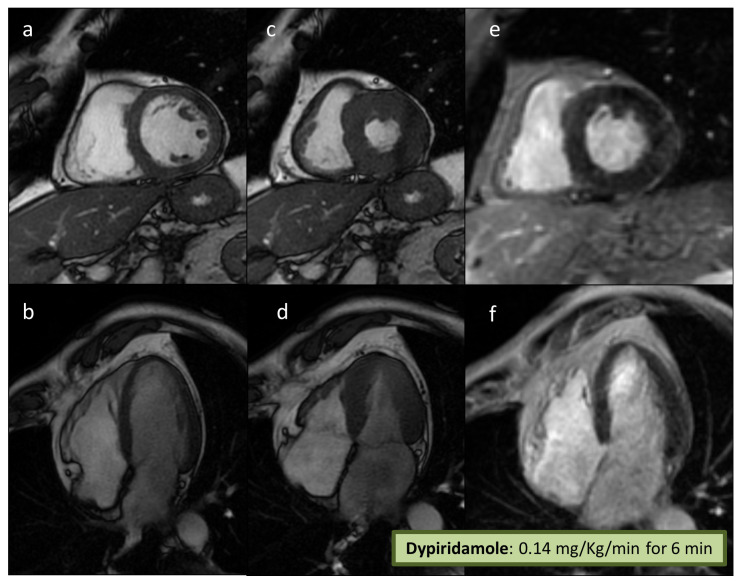
**Stress CMR.** A stress CMR was performed to exclude MI, showing normal biventricular volumes and systolic function (**a**–**d**). No LGE was detected (**e**,**f**), and a normal dynamic response was observed after dypiridamole with no signs of MI. Abbreviations: CMR = Cardiac Magnetic Resonance; MI = Myocardial Ischemia; LGE = Late Gadolinium Enhancement.

**Figure 4:**
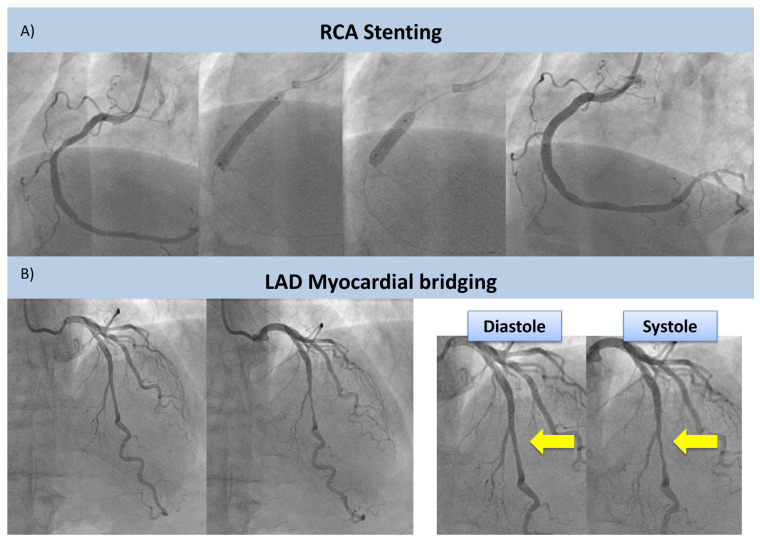
**CA and PCI.** CA shows stenosis of the RCA undergoing successful PCI without complications (upper panel, (**A**)) and the presence of a MB of the LAD (lower panel, (**B**)). MB is showed by yellow arrows. Abbreviations: CA = Coronary Angiography; RCA = Right Coronary Artery; PCI = Percutaneous Coronary Intervention; MB = Myocardial Bridging; LAD = Left Descending Artery.

A subsequent pharmacological (dipyridamole 0.14 mg/Kg/min for 6 min) stress CMR showed a normal cardiac picture (preserved biventricular function, no valvopathies, and no late-gadolinium enhancement) at rest with no induced MI (Figure 3). In view of the discrepancy between the CPET and the CMR data, a coronary angiography (CA) was performed showing single vessel coronary artery disease (CAD), with a calcified plaque on the medium right coronary artery (RCA) leading to a 70% stenosis (Figure 4A). The RCA stenosis underwent a successful percutaneous coronary intervention (PCI) without complications and with a good final angiographic result. A left descending artery (LDA) MB with a dynamic compression was also observed (Figure 4B). At two months, a follow-up same ramp maximal CPET was performed, still diagnostic for MI (Figure 2C; Table 1). The patient was treated with calcium channel blockers (Verapamil 80 mg t.d.s.), and after 2 months, another CPET was normal (no MI) (Figure 2D; Table 1).

## 3. Discussion

This clinical case report of MB-related MI demonstrates the central role of exercise (and, in particular, its evaluation by CPET) as a provocative test in patients with “dynamic” coronary artery stenosis, such as in case of MB.

Even if stress CMR is an accurate method to assess MI in patients with known or suspected CAD [11], only a few MB patients have been systematically evaluated with this technique. In these subjects, due to a pure “steal-flow” vasodilator effect without a fixed obstruction, the use of dipyridamole during stress testing may result in an underestimation of MI compared with exercise. CPET provides a unique approach to assess MI due to the direct observation of functional changes during strong physiological exercise. In these cases, especially, the use of metabolic variables on top of electrocardiographic changes allows noninvasive estimation of the impact of exercise on SV. In this case, a sudden reduction in O_2_ pulse during exercise was observed, accompanied by a decrease in VO_2_/W slope (Figure 2B,C). These findings, more than the absolute values of these parameters (which can be still considered within the normal range), suggest an abrupt reduction in left ventricular SV due to coronary stenosis of a large-caliber coronary artery or, in special cases, due to an increase in the intraventricular dynamic gradient during exercise in HCM [8]. Moreover, in the described case, the appropriateness of myocardial revascularization by PCI of the RCA is doubtful. In fact, despite an uncomplicated PCI with an excellent final result (Figure 3a), the subsequent CPET (Figure 2C) was still diagnostic for inducible ischemia, which is a sign that the treated coronary artery lesion was not responsible for the observed ischemic changes. An approach involving an invasive evaluation of coronary reserve (e.g., with Fractional Flow Reserve (FFR)) could have guided the revascularization procedure––likely avoiding it––and could have helped in the functional evaluation of the LAD MB, as previously described [12,13].

Despite the limitations associated with the use of dipyridamole, the use of CMR in the context of MB may still be of interest, especially in selected cases [14,15], e.g., in HCM-associated forms of MB [16]. In addition, a provocative approach with a positive inotropic drug (e.g., dobutamine) would have likely been effective in eliciting myocardial ischemia. However, the use of dobutamine is made more complex during CMR because it is not always well tolerated by patients [17]. Therefore, in a non-invasive setting, the use of physiological exercise could be proposed as the method of choice in MB patients, and it could eventually be associated with echocardiography or single-photon emission computed tomography.

The absolute values of the metabolic variables obtained during the CPET of this athletic subject merit further discussion. Despite the presence of clear signs of MI, the peak exercise data obtained in the first two CPETs were well within normal limits (peak VO_2_ 27.7 and 22.4 mL/kg/min, corresponding to 112% and 96% of the predicted, respectively, and peak O_2_-pulse 105% and 114% of the predicted value (Table 1)). However, in accordance with Fick’s law, which clearly demonstrates how VO_2_ values depend on peripheral extraction (and, therefore, on the level of training) as well as SV, in such a well-trained subject, one would expect even higher peak values. Confirming this, in the CPET performed after initiating medical therapy, despite the theoretical negative inotropic and chronotropic effects of the calcium antagonist, the peak VO_2_ values during exercise increased to 29.4 mL/kg/min (127% of the peak VO_2_ predicted) and the O_2_-pulse values increased to 13 mL/bpm (128% of the predicted values). These data highlight the importance of analyzing metabolic parameters not only in absolute numerical terms but also by evaluating their behavior during exercise through careful observation of the graphs.

## 4. Conclusions

We described the case of a sporty patient with MB, and, for this patient, a maximal CPET proved superior to stress CMR with dipyridamole in identifying signs of MI. This case highlights the importance of exercise in eliciting ischemia in specific cases.

## Figures and Tables

**Table 1:** CPETs data.

	Pre-PCI	Post-PCI	Post-Verapamil
**Ramp protocol (Watt/min)**	15	15	15
**VO_2_ at AT (mL/min)**	1050	884	1089
**HR at AT (bpm)**	102	83	102
**Work rate at AT (watt)**	72	60	73
**Peak VO_2_ (mL/min)**	1770	1431	1881
**Peak VO_2_ predicted (%)**	112	96	127
**Peak HR (bpm)**	154	123	145
**Peak work rate (watt)**	148	111	137
**VE/VCO_2_ slope**	25.7	25.0	24.6
**VO_2_ work slope (mL/min/Watt)**	11.3	11.5	11.9
**RR (bpm)**	27.5	24	32
**Peak VE (L/min)**	60.8	57.3	72.4
**Peak RER**	1.08	1.24	1.27

Legend: AT = Anaerobic Threshold; CPET = Cardiopulmonary Exercise Test; HR = heart rate; PCI = Percutaneous Coronary Intervention; RER = respiratory exchange ratio; RR = Respiratory Rate; VE = Ventilation; VCO_2_ = carbon dioxide consumption; VO_2_ = Oxygen Uptake.

## Data Availability

No database is available due to the nature of the study (single patient observational case report).
